# Transformation of Geniposide into Genipin by Immobilized β-Glucosidase in a Two-Phase Aqueous-Organic System

**DOI:** 10.3390/molecules16054295

**Published:** 2011-05-24

**Authors:** Yi-Shun Yang, Tong Zhang, Shai-Cheng Yu, Yue Ding, Li-Ying Zhang, Chen Qiu, Duo Jin

**Affiliations:** 1 Experiment Center of Teaching & Learning, Shanghai University of Traditional Chinese Medicine, Shanghai 201203, China; Email: becky105@163.com (Y.-S.Y.); 2 College of Chemical Engineering, East China University of Science and Technology, Shanghai 200237, China; Email: scyu@ecust.edu.cn (S.-C.Y.)

**Keywords:** immobilized β-glucosidase, geniposide, genipin, aqueous-organic two-phase system

## Abstract

Genipin is the bioactive compound of geniposide and a natural cross-linking agent. In order to improve the preparation process of genipin, the hydrolysis of geniposide to genipin by immobilized β-glucosidase in an aqueous-organic two-phase system was studied. β-Glucosidase was immobilized by the crosslinking-embedding method using sodium alginate as the carrier. The optimum reaction temperature, pH value and time were 55 °C, 4.5 and 2.5 h, respectively. To reduce genipin hydrolysis and byproduct production the reaction was carried out in an aqueous-organic two-phase system comprising ethyl acetate and sodium acetate buffer. The product was analyzed by HPLC, UV, IR, and NMR. The yield of genipin was 47.81% and its purity was over 98% (HPLC).

## 1. Introduction

Fructus Gardeniae is the dried ripe fruit of *Gardenia jasminoides* Ellis (Rubiaceae). It is widely used in Traditional Chinese Medicine because of its cholagogue, sedative, diuretic, anti-inflammatory, and antipyretic effects [1]. Geniposide, a natural iridoid glycoside, is one of the major effective compounds of Fructus Gardeniae. It has been reported that geniposide has many important curative effects, such as hepatic-protective, cholagogic effects [2], antithrombotic effects [3], and diabetes curative effects [4].

Studies have shown that geniposide has hepatic-protective and choleretic effects by oral administration, but these effects are absent if given by injection. That is because geniposide is hydrolyzed to its bioactive compound genipin by human intestinal microflora enzymes, and it is genipin that has the hepatic-protective and choleretic effects [5,6,7]. Therefore, if genipin is given directly to patients, the differences between intestinal metabolic activities of geniposide in individuals can be avoided, and the dose of genipin can be precisely controlled and this should improve its curative effects and decrease its toxicity.

**Scheme 1:**
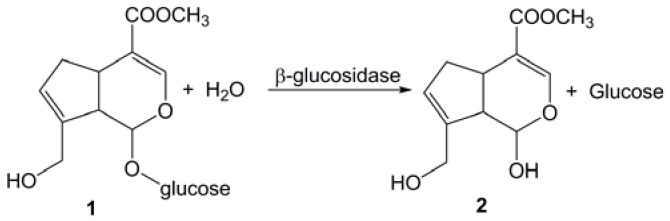
Hydrolysis of geniposide by β-glucosidase.

It is reported that genipin has many other medicinal effects, such as anti-inflammatory [5], anticancer [6], antithrombotic [3], antibacterial [7], gastritis curative [8], diabetes curative [9], neurotoxicity inhibition [10], and antidepressant-like effects [11]. In addition, genipin is a natural cross-linking agent in biological applications [12,13]. It is also used to prepare a series of blue pigments (gardenia blue) used in the food industry [14]. As the content of genipin in Fructus Gardeniae is very low [15], genipin (2) is prepared mainly from hydrolysis of geniposide (1) by β-glucosidase (Scheme 1) [16]. However, the low productivity and high product cost of this method have limited the application of genipin.

**Scheme 2:**
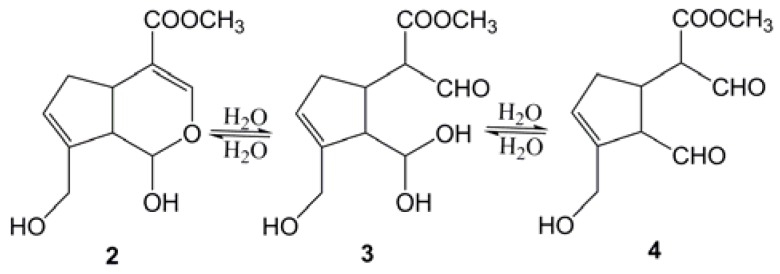
Presumed hydrolysis of genipin.

In this paper, we aimed to improve the preparation of genipin. The hydrolysis of geniposide to genipin by immobilized β-glucosidase in an aqueous-organic two-phase system was studied. Because of its hemiacetal ring-structure, genipin is unstable in water, and it probably hydrolyzes to a double-aldehyde structure 4 (Scheme 2) [17], which is similar to the cross-linking agent glutaraldehyde.

The hydrolysis of genipin decreases its yield. Therefore, an aqueous-organic two-phase system was used so the genipin produced can be extracted into the organic phase during the reaction, but the organic solvent may destroy the enzyme. Moreover, genipin crosslinks with amines, amino acids and proteins, and forms a kind of pigment (gardenia blue) [12]. As β-glucosidase is a kind of protein, genipin crosslinks with it during the hydrolysis of geniposide. Therefore, we immobilized the enzyme to protect it from both the organic solvent and genipin.

## 2. Results and Discussion

### 2.1. Optimize Immobilization Conditions of β-glucosidase by Orthogonal Experiment

Based on univariate experiments, L9 (3^4^) was used to design the orthogonal experiment. The design and results were shown in table 1.

**Table 1:** The design and results of optimizing immobilization conditions of β-glucosidase by orthogonal experiment. (A) Concentration of sodium alginate (w/v); (B) Concentration of glutaraldehyde (v/v); (C) Concentration of CaCl_2_ (w/v).

Entry	Factor			Immobilization efficiency (%)
	A (%)	B (%)	C (%)	
1	1	0.1	0.5	30.12
2	1	0.5	1	30.6
3	1	0.8	1.5	36.44
4	2	0.1	1	30.68
5	2	0.5	1.5	25.92
6	2	0.8	0.5	35.48
7	3	0.1	1.5	31.24
8	3	0.5	0.5	35.88
9	3	0.8	1	38.32
K1	32.39	30.68	33.83	
K2	30.69	30.8	33.2	
K3	35.15	36.75	31.2	
R	4.46	6.07	2.63	

As Table 1 shows, the influence was B > A > C within the range of our experiments. The optimum immobilization conditions were confirmed as below (A_3_B_3_C_1_): the sodium alginate (carrier), glutaraldehyde (cross-linking agent) and β-glucosidase were mixed to a final concentration of 3% (w/v), 0.8% (v/v) and 5 U/mL, respectively. The mixture was crosslinked in room temperature for 2 h. Then it was added dropwise to 0.5% (w/v) calcium chloride solution and left standing for 2 h. Based on the optimum immobilization conditions, the immobilization efficiency of β-glucosidase was 40.92%.

### 2.2. Optimum Reaction Temperature and pH Value

**Figure 1:**
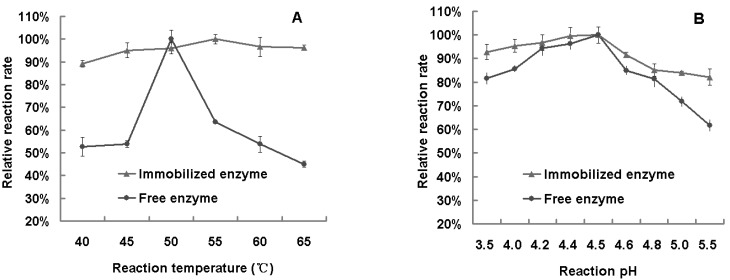
**(A)** Influence of reaction temperature to relative reaction rate. The relative reaction rate is equal to relevant reaction rate at different temperature divide by the highest reaction rate within 40~65 °C. **(B)** Influence of reaction pH value to relative reaction rate. The relative reaction rate is equal to relevant reaction rate at different pH value divide by the highest reaction rate within pH 3.5~5.5.

The reaction rate of free and immobilized β-glucosidase was assayed in different reaction temperatures (40, 45, 50, 55, 60, 65 °C). As Figure 1(A) shows, the optimum reaction temperature of free and immobilized β-glucosidase were 50 °C and 55 °C, respectively. The relative enzyme activity of immobilized β-glucosidase was obviously higher than free β-glucosidase within the temperature range of 40~65 °C. This proves that immobilization increases the heat resistance of β-glucosidase, and the immobilized enzyme can adapt to the temperature variation better than the free enzyme.

The reaction rate of free and immobilized β-glucosidase was assayed in different reaction pH values (3.5, 4.0, 4.2, 4.4, 4.5, 4.6, 4.8, 5.0, 5.5). As Figure 1(B) shows, the optimum reaction pH value of free β-glucosidase as well as immobilized β-glucosidase was 4.5. The relative enzyme activity of immobilized β-glucosidase was obviously higher than free β-glucosidase within pH value of 3.5~5.5. It proves that immobilization reduces the impact of pH changes on enzyme activity, and increases the reaction stability of β-glucosidase at different pHs and its pH tolerance.

### 2.3. Hydrolysis of Geniposide by Immobilized β-glucosidase in One-phase System

**Figure 2:**
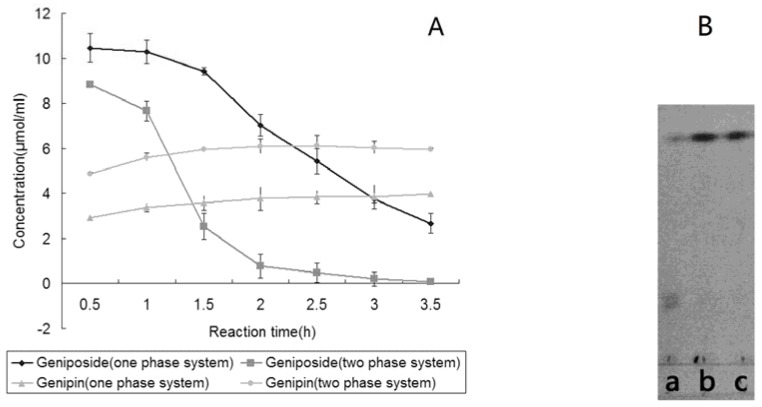
**(A)** Hydrolysis of geniposide by immobilized β-glucosidase. **(B)** TLC analysis of hydrolysis of geniposide by immobilized β-glucosidase in two-phase system. (a) Buffer layer; (b) Ethyl acetate layer; (c) Standard genipin.

The optimum reaction time was determined according to the method described in Section 3.6. As Figure 2(A) shows that when there was almost no geniposide in the reaction solution at 3.5 h, the concentration of genpin reached a peak value. Based on the above results, genipin was prepared (Section 3.8). The yield was 48 mg (31.90%). This is much lower than the theoretical yield. There were probably two reasons. First, genipin reacted with enzyme and formed gardenia blue during the reaction. We found that the reaction solution became dark blue after several hours of reaction. Secondly, genipin hydrolyzes in water, which will reduce its yield, too.

### 2.4. Hydrolysis of Geniposide by Immobilized β-glucosidase in Two-phase System

Because the yield of genipin transformed from geniposide by immobilized β-glucosidase in one-phase system was very low, a two-phase system was considered. It was comprised of 50% ethyl acetate (organic phase) and 50% sodium acetate buffer (water phase). Genipin can be extracted from the sodium acetate buffer into ethyl acetate in the two-phase system during the reaction. Thus, genipin hydrolysis and byproduct formation were reduced.

The optimum reaction time was determined according to the method described in Section 3.7. It can be seen in Figure 2(A) that the concentration of genipin was the highest when reacted for 2.5 h, while the yield was 51.16%. Based on the above results, genipin was prepared (Section 3.9). The yield was 95 mg (63.08%). The purity of genipin was over 98% (HPLC).

### 2.5. Reaction Stability of Immobilized β-glucosidase in Two-phase System

We repeated the preparation of genipin five times according to Section 3.9, and the immobilized enzyme was recycled and reused. The yields of the five repetitions were 61.69%, 57.17%, 56.71%, 47.15%, 46.61%, respectively. The yield of genipin decreased with time. There were probably three reasons. Firstly, the ethyl acetate may reduce the activity of β-glucosidase as it may destroy the spatial configuration of the enzyme. Secondly, it is known that enzyme activity decreases with time, and immobilized enzyme is no exception. Thirdly, part of enzyme reacted with genipin, and formed the gardenia blue byproduct. Therefore, the immobilized enzyme gel became blue after use, and the gel became darker when used many times. The yield of the fifth run decreased to 75.57% of that of the first time, which was in an acceptable extent.

## 3. Experimental

### 3.1. Materials

Sodium alginate (CP); glutaraldehyde (25% solution, BR); geniposide (extracted from gardeniae fruits, purity was 90% by HPLC); DNS reagent (3,5-dinitrosalicylic acid 1%, phenol 0.2%, sodium sulfite 0.05%, NaOH 1%, sodium potassium tartrate 20%); geniposide standard (NICPBP, 0749-200007) and genipin standard (Seebio Biotechnology, 6902-77-8); β-glucosidase (from *Trichoderma reesei*, enzyme activity was 867 u/g, Shanghai Baofeng biochemistry Ltd.); *p*-nitrophenyl-β-D-glucoside (*p*-NPG, Alfa Aesar).

### 3.2. Determination of β-glucosidase Activity

The activity of β-glucosidase (per unit) was defined as moles of *p*-NPG converted by g free or immobilized enzyme per minute [20]. The determination method of free β-glucosidase was as follows: free β-glucosidase (0.1 mL), 4 mM *p*-NPG solution (1 mL), sodium acetate buffer (pH 4.5, 0.9 mL) were mixed and incubated in water bath (50 °C) for 10 min. Then the reaction was stopped by adding 1 mol/L Na_2_CO_3_ solution (1 mL). The *p*-nitrophenol released was monitored at 400 nm by UV-Vis Spectrophotometer (WFZ-UV-2000, Unico Instrument, China). The activity of the immobilized β-glucosidase was determined by the same method except that 0.1 mL free enzyme solution was replaced by appropriate amount of immobilized enzyme gel and 0.1 mL sodium acetate buffer.

### 3.3. Immobilization of β-glucosidase

β-Glucosidase was immobilized by the crosslinking-embedding method using sodium alginate as the carrier [21]. Sodium alginate was dissolved in distilled water (20 mL). β-Glucosidase and cross-linking agent glutaraldehyde were added to the alginate solution and stirred immediately, then the alginate solution was added dropwise into CaCl_2_ (100 mL) from a height of 10 cm and left standing for 2 h. The gel beads were fixed. They were washed with distill water and stored in refrigerator (4 °C). 

### 3.4. Determination of Reaction Rate

The content of glucose released from hydrolysis of geniposide is equal to that of genipin (Scheme 1). Thus the reaction rate was determined as the amount of glucose released from hydrolysis of geniposide per minute:

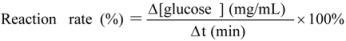
(1)

Free β-glucosidase: free β-glucosidase (0.1 mL), 4 mM geniposide solution (1 mL), sodium acetate buffer (pH 4.5, 0.9 mL) were mixed and incubated in water bath (50 °C) for 10 min. Then the reaction was stopped by adding 1 mol/L Na_2_CO_3_ solution (1 mL). DNS reagent was added for coloration [22]. Then the solution was monitored at 550 nm. Immobilized β-glucosidase: the method was the same with free enzyme except that 0.1 mL free enzyme solution was replaced by appropriate amount of immobilized enzyme and 0.1 mL sodium acetate buffer. 

### 3.5. Determination of Geniposide and Genipin

High Performance Liquid Chromatography (HPLC, Agilent) was used to determine the content of geniposide and genipin [16]. The mobile phase consists of acetonitrile and water with a ratio of 15:85 and a flow-rate of 1 mL/min. The samples were filtered by 0.45 µm microporous filtering membrane (MEMBRANA) before injection. The assay was carried out after injecting 20 µL sample to HPLC (LC-20A, Shimadzu) with the chromatographic column (Welchrom-C18, Welch) temperature of 30 °C and the detection wavelength of 238 nm.


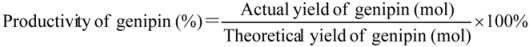
(2)

Thin layer chromatography (TLC) was used to detect geniposide and genipin [21]. Spots sampled with a capillary tube were place on GF_254_ TLC plate. The plate was dipped into a solvent comprising of ethyl acetate, chloroform, and methane acid (with the ratio of 5:4:1). Then it was detected under UV light (254 nm).

### 3.6. Determination of Optimum Reaction Time in One-phase System

Immobilized β-glucosidase (1 U) and geniposide (0.05 g) was added to sodium acetate buffer (pH 4.5, 10 mL). The mixture was incubated in water bath (50 °C) for 3.5 h. During the reaction, reaction solution (100 mL) was sampled with HPLC every half an hour to determine the concentration of geniposide and genipin.

### 3.7. Determination of Optimum Reaction Time in Two-phase System

Immobilized β-glucosidase (1 U) and geniposide (0.05 g) were added into two-phase system composed of sodium acetate buffer (pH 4.5, 10 mL) and ethyl acetate (10 mL). Then the mixture was stirred and formed an emulsion in 55 °C water bath for several hours. During the reaction, 100 μL organic phase and water phase liquid were sampled respectively with HPLC every half an hour to determine the concentration of geniposide and genipin. The total content of geniposide (or genipin) in two-phase system was equal to the sum content of geniposide (or genipin) in organic phase and water phase.

### 3.8. Preparation of Genipin by Immobilized β-glucosidase in One-phase System

Immobilized enzyme (5 U), geniposide (0.25 g) and sodium acetate buffer were mixed and incubated in 50 °C water bath for several hours. The reaction liquid was separated from immobilized enzyme gel by filtration. The solution was extracted by ethyl acetate (50 mL × 3). The above liquid (ethyl acetate) was collected and dried by anhydrous sodium sulfate. Then the liquid was concentrated to 5% (v/v) of the original volume. Put it in refrigerator (−5 °C) for 24 h and collected the crystals. The crystals were dried in a vacuum drying oven for 1h, and then recrystallized from ethyl acetate.

### 3.9. Preparation of Genipin by Immobilized β-glucosidase in Two-phase System

Immobilized β-glucosidase (5 U) and geniposide (0.25 g) were added into two-phase system composed of sodium acetate buffer (pH 4.5, 50 mL) and ethyl acetate (50 mL). Then the mixture was stirred and formed an emulsion in 55 °C water bath for several hours. The reaction liquid was separated from the immobilized enzyme gel by filtration. Then the organic phase liquid and water phase liquid were separated. The product was extracted from water phase liquid by ethyl acetate (50 mL × 2). The extract and organic phase liquid were mixed and dried by appropriate anhydrous sodium sulfate. Then the liquid was concentrated to 5% of the original volume. Put the liquid in refrigerator (−5 °C) for 24 h and collected the crystals. The crystals were dried in vacuum drying oven for 1h, and then recrystallized by ethyl acetate.

### 3.10. Identification of Genipin

The product (genipin) was analyzed by UV (TU-1901), IR (Thermo) [18], NMR (Bruker, 400 MHz) [19]. The spectral data of the product was as follows: UV (MeOH) λ_max_ 240 nm [6]. FT-IR (KBr, cm^−1^): 3398.6, 3245.7, 1681.6, 1621.9, 1443.3, 1301.2 [18]. ^1^H-NMR (CDCl_3_) δ: 7.53 (s, 3H), 5.88 (s, 7H), 4.81 (d, *J* = 8.8 Hz, 1H), 4.43~4.19 (m, 2H), 3.25~3.18 (m, 1H), 2.92~2.85 (m, 1H), 2.58~2.52 (m, 1H), 2.10~2.03 (m, 1H) ppm. ^13^C-NMR (CDCl_3_) δ: 168.17, 152.74, 142.26, 131.13, 110.96, 96.56, 95.33, 61.53, 51.55, 48.38, 39.24, 36.22 ppm [19]. The data above were in accordance with those reported before for genipin.

## 4. Conclusions

β-Glucosidase was immobilized by the crosslinking-embedding method using sodium alginate as the carrier. The optimum reaction conditions of free and immobilized β-glucosidase were studied. The optimum reaction temperature of free and immobilized β-glucosidase was 50 °C and 55 °C respectively, while for both the optimum reaction pH value was 4.5. The immobilization increased the heat resistance of β-glucosidase and reduced the impact of pH variation on its activity.

Ethyl acetate is a good solvent for genipin. In order to reduce genipin hydrolysis and byproduct formation, the hydrolysis of geniposide was carried in an aqueous-organic two-phase system comprised of ethyl acetate and sodium acetate buffer. Genipin can be extracted from sodium acetate buffer into ethyl acetate during the reaction. The optimum reaction conditions were confirmed to be as follows: a two-phase system comprising 50% ethyl acetate and 50% sodium acetate buffer (pH 4.5). The reaction temperature and time were 55 °C and 2.5 h respectively. The yield of genipin in the two-phase system reaction was higher than in one-phase system reaction. Compared with the one-phase system, the yield of genipin increased 30% and the reaction time decreased 29% in the two-phase system. The immobilized β-glucosidase can be recycled for long-term use and showed good stability in this reaction. The method of genipin preparation established in this paper is simple, easy to realize and with short production cycle. Moreover, it has solved the problem of enzyme recycling, decreased production costs, simplified the isolation and purification procedure, and laid the foundation for industrial production.
